# Socioeconomic Status Accounts for Rapidly Increasing Geographic Variation in the Incidence of Poor Fetal Growth

**DOI:** 10.3390/ijerph10072606

**Published:** 2013-06-25

**Authors:** Stephen J. Ball, Peter Jacoby, Stephen R. Zubrick

**Affiliations:** Telethon Institute for Child Health Research, Centre for Child Health Research, The University of Western Australia, P.O. Box 855, West Perth WA 6872, Australia; E-Mails: peterj@ichr.uwa.edu.au (P.J.); steve@ichr.uwa.edu.au (S.R.Z.)

**Keywords:** poor fetal growth, socioeconomic status, conditional autoregression, spatial variation

## Abstract

Fetal growth is an important risk factor for infant morbidity and mortality. In turn, socioeconomic status is a key predictor of fetal growth; however, other sociodemographic factors and environmental effects may also be important. This study modelled geographic variation in poor fetal growth after accounting for socioeconomic status, with a fixed effect for socioeconomic status and a combination of spatially-correlated and spatially-uncorrelated random effects. The dataset comprised 88,246 liveborn singletons, aggregated within suburbs in Perth, Western Australia. Low socioeconomic status was strongly associated with an increased risk of poor fetal growth. An increase in geographic variation of poor fetal growth from 1999–2001 (interquartile odds ratio among suburbs = 1.20) to 2004–2006 (interquartile odds ratio = 1.40) indicated a widening risk disparity by socioeconomic status. Low levels of residual spatial patterns strengthen the case for targeting policies and practices in areas of low socioeconomic status for improved outcomes. This study indicates an alarming increase in geographic inequalities in poor fetal growth in Perth which warrants further research into the specific aspects of socioeconomic status that act as risk factors.

## 1. Introduction

Fetal growth is a key predictor of infant health. Infants with poor fetal growth have greater risks of morbidity and mortality [1,2], birth defects [3], and poor health outcomes later in life [4]. Factors that in turn predict fetal growth may provide a basis for preventive interventions to improve birth outcomes and child development at the population level.

Much is known about the mix of potential risk factors [5]. Poor fetal growth is more likely with first births, multiple births and low levels of antenatal care [5,6,7,8]. Maternal risk factors include low socioeconomic status, smoking and alcohol consumption during pregnancy, poor fetal growth among previous pregnancies, and low maternal weight [5,6,9,10,11]. Diet, maternal age and ethnicity are also important [5,12,13]. Neighbourhood-level effects, where demonstrated beyond individual circumstance, indicate an impact of the broader social environment on fetal growth [14]. Impacts of the physical environment include effects of air pollution [15] and water contaminants [16]. Thus, a wide range of potential risk factors have been identified. The remaining challenges lie in resolving causal pathways, and in applying analytical methods to identify the relative roles of modifiable risk factors in populations of interest [17].

Area-level comparisons of disease rates help target opportunities for improved outcomes. Beyond mapping rates *per se*, interventions benefit from information on local causes of variation [18]. In any given region, an unknown combination of economic, demographic and environmental factors may influence fetal growth. Amid this uncertainty, socioeconomic status ranks highly as a candidate factor: low socioeconomic status is a widespread predictor of poor health [19,20], and has been repeatedly linked to poor birth outcomes, including poor fetal growth [5,6,8,14,21,22,23].

The motivation for this study was to model geographic variation in the incidence of poor fetal growth across urban Perth, Western Australia as an informing step for generating hypotheses about local risk factors of poor fetal growth. Given Perth’s strong geographic patterns in socioeconomic status [24,25], and previous evidence that poor fetal growth is associated with low socioeconomic status in Western Australia [21], it is likely that geographic patterns due to other processes such as environmental effects are difficult to resolve without adjusting for socioeconomic status. We therefore shifted the focus from modelling variation *per se*, to modelling the residual variation in incidence of poor fetal growth that remains while allowing for an effect of socioeconomic status. Two periods (1999–2001 and 2004–2006) were compared to characterise the temporal stability of patterns.

The value of this study is twofold. Firstly, it provides specific insights into geographic variation in poor fetal growth in Perth in terms of its relationship with socioeconomic status, and as variation unexplained by socioeconomic status. Secondly, this study explicitly models geographic variation in a health outcome after adjusting for a fixed effect. Applications of mixed models variously emphasise the benefits as decreasing the bias of fixed effect estimates [26,27,28], or in smoothing estimates of disease rates [29,30]. However, there may also be useful information about unobserved processes evident in the unexplained component of such models. With a few exceptions [31,32,33,34,35,36], the use of mixed models for this purpose is largely underutilised in health research. By adjusting for socioeconomic status and using a measure of fetal growth that adjusts for parity and maternal height (as well as gestational age and gender), this study provides a strong test for spatial patterns in poor fetal growth beyond a socioeconomic effect.

## 2. Methods

### 2.1. Study Design and Setting

This was a time-stratified study of spatial patterns in the incidence of poor fetal growth among liveborn singleton neonates in urban Perth, Western Australia. The data were analysed separately for each of two periods: 1999–2001 and 2004–2006.

### 2.2. Data Source

**Figure 1:**
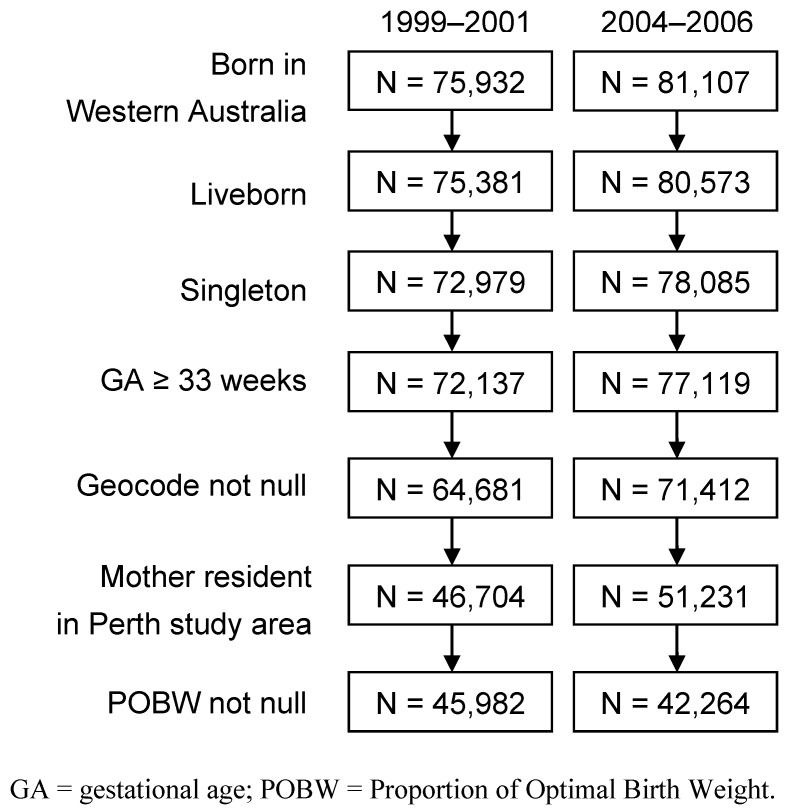
Selection of records for the two study periods.

Birth data were sourced from the Western Australian Maternal and Child Health Research Database, a population-wide database of all children born in Western Australia since 1980 [37]. The data were restricted to liveborn singletons, and excluded births less than 33 weeks gestation (Figure 1) because our measure of poor fetal growth is potentially less accurate at earlier ages [38].

### 2.3. Spatial Units

The spatial units were suburbs [39]. Of the 297 suburbs in Perth in 2006, seven were excluded from analysis for having more than 50% of their area outside the urban area, 20 were excluded for having no births in at least one of 1999–2001 and 2004–2006, and three were excluded because socioeconomic data were unavailable. Hereafter the term “study area” refers to the 267 suburbs used for analysis.

### 2.4. Definition of Poor Fetal Growth

We used a standardised measure of fetal growth called the Proportion of Optimal Birth Weight, POBW [38]. POBW is the ratio of an infant’s actual birth weight relative to the weight expected from their combination of gestational duration, fetal gender, maternal height and parity [38]. Thus, at the same time as explicitly adjusting for socioeconomic status in this study, POBW adjusts for these other variables that comprise the POBW calculation. Every birth under 80% Optimal Birth Weight was categorised as having poor fetal growth. Preliminary analysis showed that this threshold classified 5% of live singleton births as having poor fetal growth, which we saw as a reasonable compromise between effect size and number of cases. Records that were null for POBW were excluded from the analysis. Ascertainment of POBW dropped from 98.5% in 1999–2001 to 82.5% in 2004–2006 (Figure 1), largely due to decreasing compliance in measuring maternal height (A. Langridge, personal communication, 2013). It is assumed that non-ascertainment had a negligible impact on the estimated effect size of socioeconomic status and on random effects estimates. These assumptions were tested by examining small-for-gestational-age (SGA) among records in 2004–2006 that were null for POBW. SGA provides an alternative measure of poor fetal growth which is unadjusted for maternal height and parity. SGA was calculated using national centiles of birthweight, stratified by sex [40]. While SGA was more likely among records null for POBW (odds ratio = 1.31, confidence interval 1.17 to 1.46), there was no significant difference in the odds ratio among quintiles of socioeconomic status (χ^2^ = 6.81, d.f. = 4; Breslow-Day Test of Homogeneity *p*-value = 0.15). Furthermore, among records null for POBW in 2004–2006, the distribution of cases of SGA among suburbs was consistent with probabilities of low POBW estimated from the random effects model (χ^2^ = 266.70, d.f. = 254, *p* = 0.28).

### 2.5. Socioeconomic Status

Socioeconomic status was based on the Australian Bureau of Statistics’ Index of Relative Socio-economic Advantage and Disadvantage [24,25], hereafter shortened to the “Advantage-Disadvantage Index”. This is one of four area-level socioeconomic indices derived by the Bureau from principal component analysis of area-level summaries of individual, family, and household data collected during each 5-year national census. The Advantage-Disadvantage Index focuses on the presence of both positive and negative social and economic factors. Variables include indicators of income, employment status, class of work (e.g., professional, labourer, machinery-operator), education status, car ownership, internet access, monthly rent or mortgage payments, number of rooms per dwelling, and family structure. We used the 2001 and 2006 versions of the Index respectively to model the 1999–2001 and 2004–2006 data. There is a strong overlap in the list of variables between these censuses [24,25]. While the Index is standardised nationally, it was standardised to a mean of 0 and standard deviation of 1 within the study area for each study period.

### 2.6. Models of Poor Fetal Growth

The incidence of poor fetal growth was modelled using logistic regression. The null model specified all suburbs as having the same probability of poor fetal growth:

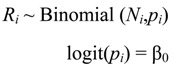
(1)
where *R_i_*, *N_i_* and *p_i_* are respectively the number of poor fetal growth births, total number of births, and probability (per birth) of poor fetal growth in suburb *i*. The constant β_0_ is the log odds of the mean probability of poor fetal growth.

The random effects model allowed for variation in the probability of poor fetal growth among suburbs:

logit(*p_i_*) = β_0_ + *u_i_* + *v_i_* (2)

where *u_i_* is a spatially-correlated random effect and *v_i_* is a spatially-uncorrelated random effect, following Besag *et al.* [41]. A conditional autoregressive (CAR) term was used for the spatially-correlated random effect, which is specified as following a normal distribution with a mean of zero relative to, or conditional on, the mean CAR random effect estimates of neighbouring areas [42]. The uncorrelated random effect was specified as being normally-distributed with a mean of zero, and no constraint of correlation among neighbouring areas.

The fixed effect model treated the variation among suburbs as a function of socioeconomic status, in the absence of random effects:

logit(*p_i_*) = β_0_ + β_1_·*X_i_* (3)

where *X_i_* is the standardised Advantage-Disadvantage Index of suburb *i*.

The full (mixed) model, combining the random effects in Model (2) with the fixed effect in Model (3), was used to explicitly model variation in the probability of poor fetal growth while accounting for socioeconomic status:

logit(*p_i_*) = β_0_ + β_1_·*X_i_* + *u_i_* + *v_i_* (4)

This model enabled measurement of the effect of socioeconomic status (through the constant, β_1_), while simultaneously characterising spatially-correlated and uncorrelated extra-binomial variation in the data through the standard deviation of *u* and *v*. Based on this full model, suburb-specific values of *u_i_* and *v_i_* estimate geographic variation in poor fetal growth after adjustment for socioeconomic status.

The four models were analysed separately for the 1999–2001 and 2004–2006 datasets to allow for changing patterns over time. Each model was run as a Bayesian analysis with WinBUGS 1.4 software [43], which uses Markov Chain Monte Carlo (MCMC) sampling to generate posterior distributions. Uninformative (*i.e.*, widely dispersed) normal prior distributions were used for each of β_0_ and β_1_ (mean = 0; standard deviation = 100). The standard deviation of the spatially-correlated and uncorrelated random effects were each specified as having an uninformative half-normal prior distribution with mean of 0 and standard deviation of 100, following Gelman [44].

Modelling of the spatially-correlated random effect required a matrix of suburb adjacencies. Each suburb’s neighbours were defined as those suburbs with one or more common boundaries or vertices with the suburb in question, generated using SpaceStat 2.2 software. Supplementary adjacencies were used to join suburbs across the Swan River estuary wherever suburbs were less than 1km apart. Supplementations were similarly assigned across opposite sides of the Canning River, a southern tributary to the Swan River estuary.

Relative support for different model scenarios was assessed using the Deviance Information Criterion, DIC [42,45]. The DIC measures how well a model fits the observed data, while adding a penalty for additional parameters.

Preliminary analyses showed high levels of autocorrelation between MCMC samples. Sampling was therefore thinned to one in every 100 simulations. Posterior distributions were generated from 1,000,000 simulations (*i.e.*, 10,000 thinned samples) after an initial burn-in of 1,000,000 simulations. Convergence was confirmed using the Geweke diagnostic [46].

### 2.7. Additional Software

All maps were generated using ArcGIS Desktop 9.2.

## 3. Results

The mean incidence of poor fetal growth was very similar in 1999–2001 and 2004–2006. There were 2,194 cases of poor fetal growth from 45,982 births in 1999–2001 (incidence = 4.8%), and 1,993 cases from 42,264 births in 2004–2006 (incidence = 4.7%). However, this is likely to be a slight underestimate of the 2004–2006 rate, given the higher rate of missing data in that period that had a bias towards smaller births (section 2.4).

**Figure 2:**
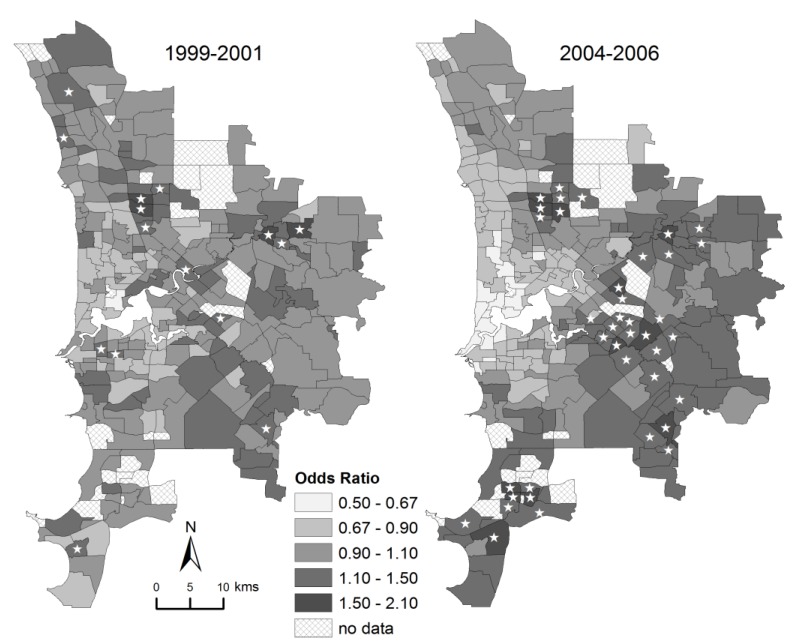
Mapped variation in the incidence of poor fetal growth in Perth in 1999–2001 and 2004–2006. This shows the odds ratio of poor fetal growth relative to the mean incidence, calculated from the sum of spatially-correlated and uncorrelated random effects from the random effects model. Stars denote suburbs with a posterior probability greater than 0.90 of the odds ratio exceeding 1.0 relative to the mean incidence.

There was strong variation among suburbs in the probability of poor fetal growth (Figure 2). This variation increased from having an interquartile odds ratio among suburbs of 1.20 in 1999–2001 to 1.40 in 2004–2006 (Table 1).

**Table 1:** Effect sizes for random effects and socioeconomic status, expressed as interquartile odds ratios among suburbs in poor fetal growth.

Period	Model	Effect(s)	IQOR ^a^
1999–2001	2. Random effects	Spatially-uncorrelated random effect	1.13
		Spatially-correlated random effect	1.06
		Combined random effects	1.20
	4. Full model	Spatially-uncorrelated random effect	1.07
		Spatially-correlated random effect	1.03
		Combined random effects	1.09
		Socioeconomic status	1.41
2004–2006	2. Random effects	Spatially-uncorrelated random effect	1.03
		Spatially-correlated random effect	1.40
		Combined random effects	1.40
	4. Full model	Spatially-uncorrelated random effect	1.04
		Spatially-correlated random effect	1.06
		Combined random effects	1.09
		Socioeconomic status	1.46

^a^ IQOR (interquartile odds ratio) was calculated as the ratio of the odds of poor fetal growth of the 75th centile among suburbs relative to the 25th centile. The source data for each IQOR was the mean effect size per suburb (*i.e.*, mean output of Markov Chain Monte Carlo simulations) of the 267 suburbs. The effect of socioeconomic status is also reported as an interquartile odds ratio for comparison with random effects, despite modelling it as a fixed effect.

The statistical importance of this variation was confirmed by the improvement in model fit by adding random effects to the null model, whereby the DIC decreased by 60.0 in 1999–2001, and 117.5 in 2004–2006 (Table 2).

**Table 2:** Summary of model diagnostics: Deviance Information Criterion (DIC), effective number of parameters (pD), mean probability of poor fetal growth (*p*), and the slope parameter (β_1_) for the socioeconomic effect (this measures the rate of change in the log odds of poor fetal growth for an increase of one standard deviation in the Advantage-Disadvantage Index). Values in brackets delimit Bayesian 95% credible intervals.

Period	Model	DIC	pD	*p*	β_1_
1999−2001	1. Null model	1,277.3	1.0	0.050 (0.048, 0.052)	
	2. Random effects	1,217.3	81.2	0.047 (0.045, 0.050)	
	3. Fixed effect	1,195.4	2.0	0.050 (0.030, 0.087)	−0.21 (−0.25, −0.16)
	4. Full model	1,180.5	48.7	0.049 (0.025, 0.098)	−0.22 (−0.28, −0.17)
2004−2006	1. Null model	1,320.3	1.0	0.049 (0.047, 0.052)	
	2. Random effects	1,202.8	72.7	0.047 (0.045, 0.050)	
	3. Fixed effect	1,183.3	2.0	0.052 (0.028, 0.097)	−0.27 (−0.32, −0.23)
	4. Full model	1,176.5	38.0	0.052 (0.022, 0.111)	−0.26 (−0.31, −0.19)

The incidence of poor fetal growth was spatially structured in both periods. Oden’s test of association [47] (generated using ClusterSeer 2.3 software) showed strong evidence of a spatial pattern between adjacent suburbs in 1999–2001 (I_pop_ = 0.031; *p* < 0.001) and in 2004–2006 (I_pop_ = 0.066; *p* < 0.001). Furthermore, the random effects model indicated that spatial structure was much stronger in 2004–2006 than in 1999–2001 (Table 1). In 2004–2006 the spatially-correlated random effect accounted for 87.2% of the total variance, while it accounted for 26.5% of the variance in 1999–2001 (calculated as the mean across MCMC samples of σ*^2^_u_*/σ*^2^_u+v_* from the random effects model).

**Figure 3:**
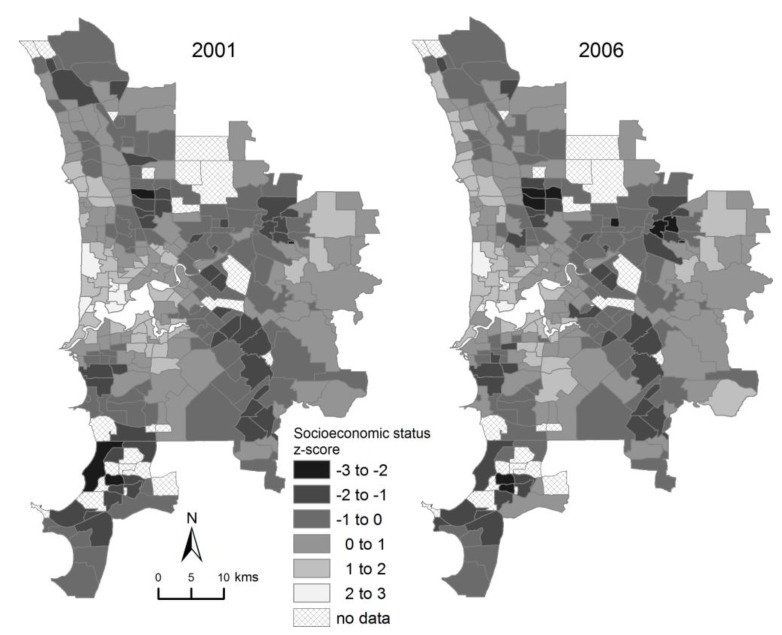
Socioeconomic variation in Perth, mapped as standard deviations from the mean Advantage-Disadvantage Index in each of 2001 and 2006.

Socioeconomic status was spatially structured in both periods (Figure 3). Moran’s I test [48] (generated using ClusterSeer 2.3 software) indicated strong evidence of positive autocorrelation in socioeconomic status between adjacent suburbs (I = +0.59, *p* < 0.001 in 1999–2001; I = +0.53, *p* < 0.001 in 2004–2006). Socioeconomic status was highly conserved between the two periods: the suburb-by-suburb Pearson correlation between the two periods was +0.95.

There was a strong negative relationship between socioeconomic status and the probability of poor fetal growth in both periods (Table 2). Thus, on average, suburbs with higher socioeconomic status had a lower probability of poor fetal growth. The gradient between socioeconomic status and probability of poor fetal growth was steeper in 2004–2006 than in 1999–2001. The importance of socioeconomic status as an explanatory variable is indicated by the improved level of model fit achieved by adding socioeconomic status to the null model and random effects model. The model regression coefficients were weakly sensitive to the inclusion of the random effects.

**Figure 4:**
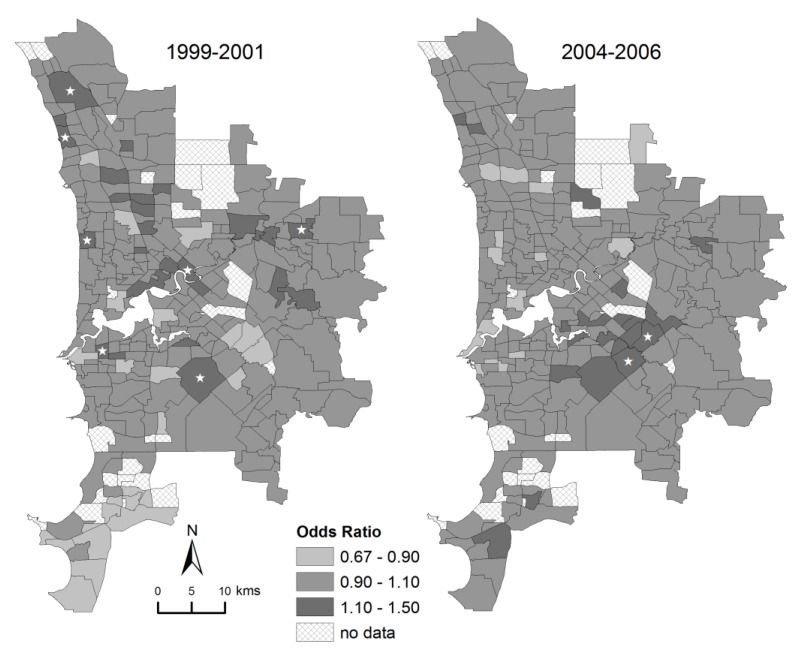
Mapped residual variation in the incidence of poor fetal growth in Perth after accounting for socioeconomic status. This shows the odds ratio of poor fetal growth, calculated from the sum of spatially-correlated and uncorrelated random effects from the full (mixed) model. Stars denote suburbs with a posterior probability greater than 0.90 of the odds ratio exceeding 1.0 relative to the socioeconomic-adjusted mean.

In both periods, the best model of variation among suburbs in the probability of poor fetal growth was the full model that included socioeconomic status and random effects. Adding random effects to the fixed effect model decreased the DIC by 14.9 in 1999–2001, and by 6.8 in 2004–2006 (Table 2). Furthermore, in the full model, some suburbs had a high probability (>0.90) of exceeding the socioeconomic-adjusted mean incidence of poor fetal growth (seven suburbs in 1999–2001; two suburbs in 2004–2006; see Figure 4).

While random effects improved the level of model fit after accounting for socioeconomic status, the magnitude of residual variation was low in both periods (Figure 4). Compared to the strong fixed effect of socioeconomic status which accounted for an interquartile odds ratio among suburbs of 1.41 in 1999–2001 and 1.46 in 2004–2006, the interquartile odds ratio for the combined random effects was 1.09 in both periods (Table 1).

The random effects estimated after accounting for socioeconomic status were inconsistent over time. There was a negative relationship in the suburb-by-suburb Pearson correlation of spatial random effects in 1999–2001 *versus* 2004–2006 (r = −0.48). This corresponded to a switch in the geographic gradient of the spatially-correlated random effect, whereby the odds of poor fetal growth increased from south-to-north in 1999–2001, but increased from northwest-to-southeast in 2004–2006 (pattern not shown). The uncorrelated random effect showed very low stability, with no clear relationship in random effects between 2004–2006 and 1999–2001 (r = +0.07).

## 4. Discussion

We expected to observe a strong and persistent geographic pattern in the incidence of poor fetal growth in Perth after taking socioeconomic status into account, but didn’t. Socioeconomic status was initially identified as one of many possible causes for geographic variation in fetal growth so that after accounting for socioeconomic status a strong residual pattern was expected from other, unmeasured factors. Such a result was reported in Northern Ireland [35]. While the random effects in our study improved the level of model fit in both periods, the effect size of this variation was both small in magnitude and unstable over time. This result contrasts with evidence that variation in fetal growth in Perth is spatially associated with traffic pollution [15]. It therefore seems that the effects of traffic pollution, and any other environmental causes for variation in fetal growth in Perth over the study period were relatively small in magnitude or operate at such a small spatial scale that their variation is effectively averaged at the scale of suburbs.

Instability in the spatially-correlated component of residual variation occurred as a switch in geographic pattern, with the risk of poor fetal growth increasing south-to-north in 1999–2001 and increasing from northwest-to-southeast in 2004-2006. Uncorrelated residual variation among suburbs, effectively the ‘suburb effect’ after accounting for socioeconomic status, was also unstable through time. Any proposal of what may be causing residual variation needs to account for these patterns of instability. In the absence of environmental candidates for these changes, we propose that the patterns of residual variation may be caused by shifting spatial patterns of sociodemographic composition not captured by the index used to model socioeconomic status. Such changes are possible within the context of the sustained resources boom in Perth over the study period, which was accompanied by rapid social, demographic and economic change [49]. Furthermore, it seems unlikely that changes in spatial patterns of the random effects were artefacts of measurement error in the socioeconomic index, given that the index was so highly correlated between the two periods.

While the motivation for this study was to characterise variation beyond the effects of socioeconomic status, there was a very strong socioeconomic effect in both periods. Ultimately any effect of an external risk factor on fetal growth must occur as a physiological impact via the placenta. As an indicator of social position, socioeconomic status itself cannot have such an effect; other mediating mechanisms must act as the proximal causes [50]. A logical follow-up to this study would be to unpack the specific factors which act as mediators of a socioeconomic association with poor fetal growth in Perth. These could include smoking, alcohol consumption, maternal weight, maternal age, diet, poorer antenatal care and neighbourhood effects [5,8,9,10,12,14]. There may also be environmental conditions correlated with socioeconomic status which impact fetal growth.

Two strong features of the data were: (a) that the overall level of variation among suburbs in the probability of poor fetal growth increased from 1999–2001 to 2004–2006, and (b) that this variation became more spatially structured over the same period. An initial consideration was that these changes in variation and spatial structure of poor fetal growth may be attributable to increased variation and spatial structure in socioeconomic status; however this was not the case. Socioeconomic status became less spatially structured in 2004–2006, as indicated by Moran’s I statistic. Nonetheless, socioeconomic status played a stronger role in explaining variation between suburbs in 2004–2006 than in 1999–2001, with the result that after accounting for socioeconomic status, the level of residual variation in poor fetal growth was similarly low in both periods. Given that the relationship between socioeconomic status and poor fetal growth was steeper in 2004–2006 than in 1999–2001, it follows that the strengthening of the fixed effect of socioeconomic status was responsible for the observed increase in geographic variation of poor fetal growth.

The results of this study are consistent with previous evidence of a steepening socioeconomic gradient in poor fetal growth over a similar period in Western Australia [21] and elsewhere in Australia [8]. Additionally, this study demonstrated a rapid increase in geographic disparities in the incidence of poor fetal growth, despite minimal changes in the spatial configuration of socioeconomic status. Given that these changes coincided with Perth’s sustained economic boom [49], this presents an example of how geographic variation in health outcomes can increase despite a region’s increasing economic prosperity. Rather than leading to a population-wide improvement in health, regional prosperity may in some situations fuel health inequalities, possibly by driving greater differences in income and access to health care.

Our results identify socioeconomic status as a dominant predictor of geographic clustering of poor fetal growth in Perth. This study is unique in simultaneously: (a) measuring fetal growth in a way that adjusts for parity and maternal height (as well as gestational age and gender), and (b) adjusting for socioeconomic status in a mixed model that explicitly models residual variation as a set of random effects. In combination, this provides a strong test for spatial patterns in poor fetal growth beyond a socioeconomic effect. The low levels of residual spatial patterns strengthen the case for targeting policies and practices in areas of low socioeconomic status for improved outcomes. By sharpening the focus on socioeconomic status as a strong predictor of geographic variation in poor fetal growth, the results of this study help justify the next step of unpacking the socioeconomic effect to identify the locally-important risk factors that are causal and modifiable.

## 5. Conclusions

This study identified rapidly-increasing geographic variation in the incidence of poor fetal growth in Perth. Low levels of residual variation and a strong effect of socioeconomic status suggest that socioeconomic status accounts predominantly for the observed variation. These results indicate an alarming increase in geographic inequalities in poor fetal growth in Perth which warrants further research into the specific aspects of socioeconomic status that act as risk factors.
